# Vincristine Promotes Transdifferentiation of Fibroblasts Into Myofibroblasts *via* P38 and ERK Signal Pathways

**DOI:** 10.3389/fphar.2022.901000

**Published:** 2022-05-09

**Authors:** Hui Xu, Jingwen Yang, Mengyun Tu, Jie Weng, Mengying Xie, Zhiliang Zhou, Peisen Zhou, Liang Wang, Chan Chen, Zhiyi Wang

**Affiliations:** ^1^ Department of General Practice, The Second Affiliated Hospital of Wenzhou Medical University, Wenzhou, China; ^2^ Department of Geriatric Medicine, The First Affiliated Hospital of Wenzhou Medical University, Wenzhou, China; ^3^ Department of Clinical Laboratory, Hangzhou TCM Hospital Affiliated to Zhejiang Chinese Medical University, Hangzhou, China; ^4^ Department of Emergency Medicine, The Second Affiliated Hospital of Wenzhou Medical University, Wenzhou, China; ^5^ Department of Public Health, Robbins College of Health and Human Sciences, Baylor University, Waco, TX, United States; ^6^ Institute of Bioscaffold Transplantation and Immunology, School of Basic Medical Sciences, Wenzhou Medical University, Wenzhou, China; ^7^ Institute of Hypoxia Medicine, School of Basic Medical Sciences, Wenzhou Medical University, Wenzhou, China

**Keywords:** vincristine, pulmonary fibrosis, fibroblast, myofibroblast, MAPK

## Abstract

**Background:** Vincristine (VCR) is used in the clinic as an anti-tumor drug. VCR can cause pulmonary fibrosis (PF), leading to respiratory failure. The transformation of fibroblasts into myofibroblasts may play a key role in PF. The present study attempted to reveal the molecular mechanism of VCR-induced PF and the possible involvement of the mitogen-activated protein kinase (MAPK) signaling pathway.

**Methods:** Human embryonic lung fibroblasts (HELFs) were treated with different concentrations of VCR. Inhibitors of extracellular signal-regulated kinase 1/2 (ERK1/2) and p38 MAPK were added to HELFs. Cell proliferation state was assessed using cell counting kit-8 and by directly counting the number of cells. The expressions of vimentin and *α*-smooth muscle actin (α-SMA) were investigated using western blot and immunofluorescence analyses. Activation of ERK and P38 was estimated by the expression of phosphorylated p38 MAPK (p-p38), p38 MAPK, phosphorylated ERK1/2 (p-ERK1/2) and ERK1/2 using western blot analysis. Enzyme-linked immunosorbent assay was used to estimate the level of collagen I in cell culture supernatants.

**Results:** Results showed that VCR promoted cellular proliferation, secretion of collagen I and the expression of vimentin and *α*-SMA. High expression of p-p38 and p-ERK1/2 was associated with the activation of the MAPK signaling pathway. MAPK inhibitors SB203580 and PD98059 suppressed the expression of the above proteins.

**Conclusion:** Our study revealed that VCR could promote the differentiation of fibroblasts into myofibroblasts by regulating the MAPK signal pathway, which may be a promising way to treat VCR-induced PF.

## Introduction

Pulmonary fibrosis (PF) is a life-threatening pulmonary disease with multiple causes, including environmental factors (such as inhalation of particulates) (Biondini et al., 2020; Türkkan et al., 2021), various kinds of diseases ((such as the COVID-19 pandemic, Antisynthetase syndrome) (Baratella et al., 2021; Orlandi et al., 2021) and adverse effects of specific drugs (Wang et al., 2015; Wan et al., 2020; Weng et al., 2020). Some specific drugs, especially for carcinoma, can lead to PF, which is one of the major causes of death worldwide (Xu et al., 2021). Vincristine (VCR) is an alkaloid extracted from *Catharanthus roseus* of the Apocynaceae family. It is effective in treating acute lymphoblastic leukemia, Hodgkin’s disease, lymphosarcoma and breast cancer (Okamoto et al., 2016; Yao et al., 2019). However, one of the most important adverse effects is PF. Although several studies have shown that VCR-induced PF may be associated with oxidative stress, inflammation or pulmonary epithelial cells, the underlying mechanism remains elusive (K. Zhang & Xu, 2020). Therefore, exploring the exact mechanism of VCR-induced PF will be of significant clinical importance.

Fibroblasts (FB) play a very essential role in the process of PF (H. Zhao et al., 2020). In pathological conditions, FBs are involved in the process of PF mainly through their abnormal proliferation and transformation and secretion of large amounts of extracellular matrix (ECM) (Chanda et al., 2019; Y.; Zhang et al., 2019). Moreover, several factors, especially the stimulation of a large number of cytokines, can cause FBs proliferation and transformation into myofibroblast (MB) during PF, leading to the secretion of a large amount of ECM. The MBs express *α*-smooth muscle actin (α-SMA), which is considered to be a crucial marker for MB (Xie et al., 2015; L.; Zhang et al., 2020).

Accumulating evidence has proved that MB differentiation is related to the pathogenesis of PF (Wei et al., 2019; Li N et al., 2020). MBs proliferate continuously, the survival time of single cells is greatly prolonged and a large number of collagen fibers are produced, which indicate MBs are important promoters of PF. Some studies showed that the mRNA expression of type I collagen increased in a-SMA positive cells, which proved that MBs were the key source of collagen gene expression. These pieces of evidence suggest that MBs are the primary cell types causing abnormal deposition of ECM. MBs can also accelerate the course of fibrosis by aggravating the epithelial injury and inflammatory reaction (Wei et al., 2019; Andugulapati et al., 2020).

Thus, inhibiting the transformation of lung FBs into MBs may be a promising strategy to treat VCR-induced PF. The signaling pathway of p38 and extracellular signal-regulated kinase (ERK) are considered to play an important role in cell proliferation and differentiation (Sun et al., 2015; Lavoie et al., 2020). Previous studies showed that p38 and ERK participated in the process of PF (Weng et al., 2019; Yu et al., 2019). Here, we attempted to reveal the molecular mechanism of VCR-induced PF and the potential effect of mitogen-activated protein kinase (MAPK) signaling pathway.

## Materials and Methods

### Antibodies and Reagents

VCR injection was provided by Selleck Corporation (Beijing, China). Human embryonic lung fibroblasts (HELFs) were obtained from the Shanghai Cell Bank of the Chinese Academy of Sciences (Shanghai, China). Antibodies against vimentin, *α*-SMA, p-ERK, ERK, p-p38MAPK and p38MAPK were purchased from Cell Signaling Technologies (Boston, MASS, United States). Collagen I concentration was determined by the enzyme-linked immunosorbent assay (ELISA) kit (Abcam, Cambridge, UK). Cell counting kit -8 (CCK-8) was acquired from Abcam (Cambridge, UK). Antibodies to GAPDH were purchased from Proteintech (Wuhan, China).

### Cell Culture and Treatment

HELFs were cultured in Eagles minimum essential medium (Corning Inc., Corning, NY, United States) supplemented with 10% fetal calf serum (FCS), streptomycin and penicillin (both 100 U/ml) under a 5% CO2-containing humidified atmosphere at 37°C. Cells were subcultured in T-flasks about every 5 days (Corning, United States). After washing twice with 10 ml of phosphate-buffered saline (PBS) and treating with trypsin (0.25%) and EDTA (0.02%) PBS, cells were removed from flasks after reaching 80–90% confluence. Cells were resuspended in a culture medium after incubating for 5 min at 37°C and seeded on a dish at a density of 4 × 106 cells/100-mm. HELFs were treated with different concentrations of VCR (0, 0.35, 0.7 and 1.4 μg/ml) for 24 h after overnight serum starvation.

### Cell Proliferation Assay

HELFs (1 × 104 cells/well) were maintained in a complete culture medium in 96-well plates until they reached 60–70% confluence. Cells were incubated with CCK8 solution (10 μL) at 37°C for 3 h in the presence or absence of SB203580 or PD98059 (MedChemExpress, United States) after stimulation with VCR. The cell proliferation rate was then determined by measuring the absorbance at a wavelength of 450 nm using a microplate reader.

### ELISA

As an indicator of PF, the amount of collagen I in cell culture supernatants was measured using commercial ELISA kits. Supernatants were collected after centrifugation (2500 × g) at 4°C for 10 min. All of the samples were measured in triplicate, according to the manufacturer’s instructions.

### Western Blotting Analysis

Total cell protein was extracted by radioimmunoprecipitation assay (RIPA) buffer, and the protein concentration was detected using the Bicinchoninic Protein Assay kit (BCA, Beyotime Biotechnology, China). The cells were washed thrice in PBS and then re-suspended in cold RIPA buffer. The cell lysates were centrifuged at 12,000 × g at 4°C for 15 min, and supernatants were collected. Total proteins (5–10 μg) were exposed to 10% sodium dodecyl sulfate-polyacrylamide gel electrophoresis (SDS-PAGE, Beyotime Biotechnology, China) and then transferred to nitrocellulose membranes (Pall Corporation, United States). Next, the membranes were sealed for 2 h with 5% fat-free milk at room temperature, and then co-incubated overnight at 4°C with primary antibodies: anti-p-ERK1/2, anti-ERK1/2, anti-p-p38, anti-p38, anti-vimentin and anti-α-SMA. The membranes were washed and then incubated with secondary antibody goat anti-rabbit IgG at room temperature for 1 h. An anti-GAPDH antibody was used as the internal reference. Signals were detected using an enhanced chemiluminescence-detecting kit according to the manufacturer’s protocol (Bio-Rad Laboratories, Hercules, CA, United States). The band intensity was analyzed using ImageJ software (National Institutes of Health, Bethesda, MD, United States).

### Immunofluorescence Analysis

Cells were fixed with 4% paraformaldehyde at room temperature for 20 min. Cells were then treated with 0.3% Triton X-100 for 6 min. Non-specific staining was prevented using 5% goat serum. Cells were incubated with combinations of primary antibodies: anti-vimentin or anti-α-SMA at 4°C overnight in a humidified chamber. Cells were washed thrice and then cultured with combinations of secondary antibodies for 1 h. After washing with PBS, cell nuclei were stained with 4,6-diamidino-2-phenylindole (DAPI). The sections were cover-slipped and then examined using a fluorescence microscope (Leica Microsystems, Wetzlar, Germany).

### Statistical Analysis

SPSS 19.0 statistical software (Armonk, New York: IBM Corp.) was used for data analysis. All data were presented as the mean ± SEM from at least three independent experiments. Comparisons between groups were performed by one-way analysis of variance (ANOVA) and Tukey’s multiple comparison test. *p* < 0.05 was considered statistically significant.

## Results

### VCR Induces HELF Proliferation in a Concentration-Dependent Manner

**FIGURE 1:**
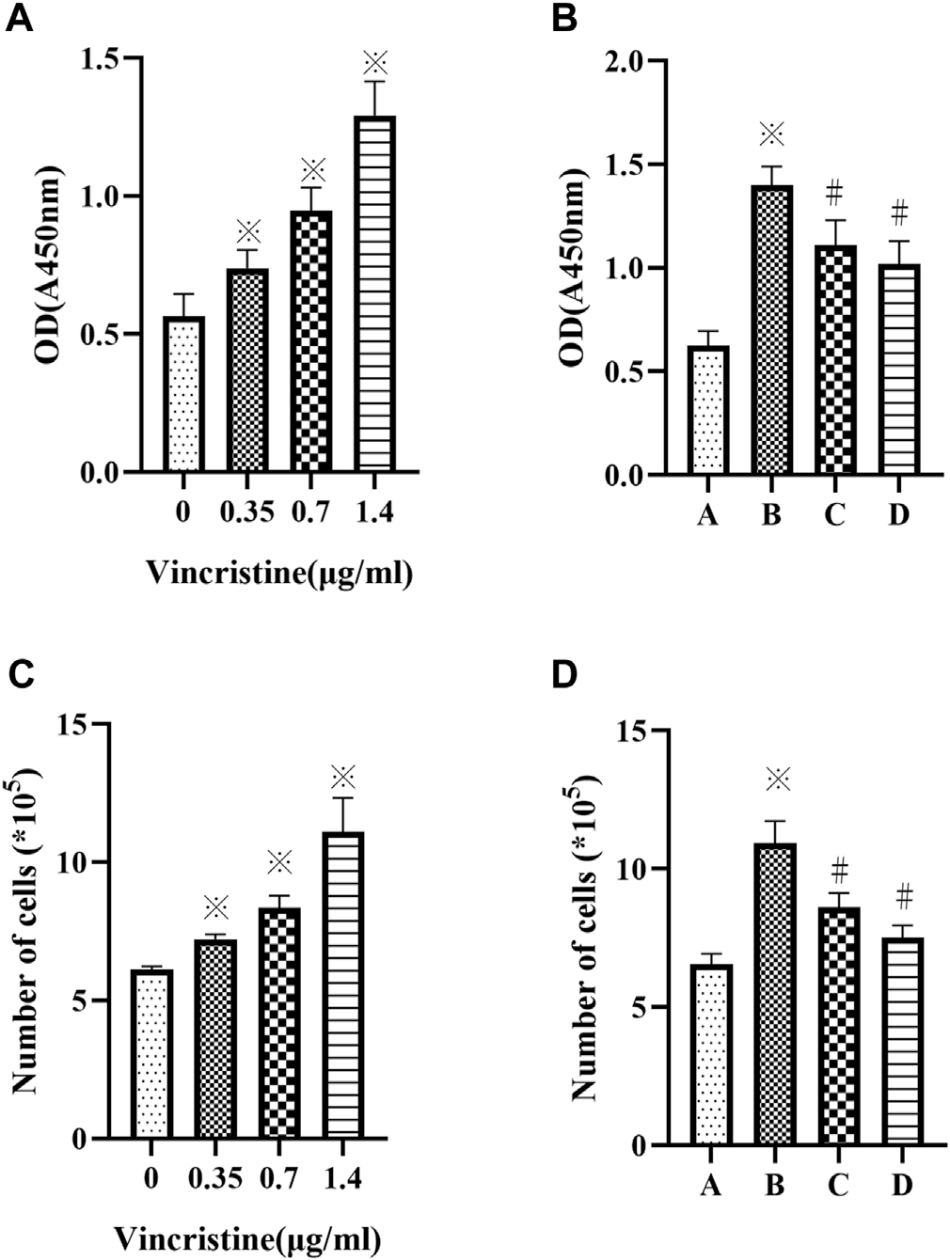
**(A,C)**: Vincristine (VCR) increases cell proliferation in HELFs. Cells were treated with different concentration of vincristine (0, 0.35, 0.7 and 1.4 μg/ml) for 24 h *p < 0.05 *vs.* the control group. **(B,D)**: VCR-induced cells proliferation was decreased by SB203580 and PD98059 in HELFs (cells were treated with 1.4 μg/ml VCR for 24 h). Cell proliferation was assessed by CCK8 assay. *p < 0.05 *vs*. the control group; #*p* < 0.05 *vs*. the VCR group. A: control group, B: VCR group, C: SB203580 group, D: PD98059 group.

As VCR was observed to be cytotoxic *in vitro* at high concentrations (J. E. Sun et al., 2016). The proliferation of HELFs treated with different concentrations of VCR (0, 0.35, 0.7 and 1.4 μg/ml) for 24 h was assessed using CCK-8 assay. The number of cells was also counted. Results showed that compared with the control group, VCR treatment significantly increased HELF proliferation in a concentration-dependent manner (F = 71.18, *p* < 0.05 and F = 62.98, *p* < 0.05 respectively) (Figures 1A,C). HELF proliferation was the most significant at a concentration of 1.4 μg/ml. Therefore, 1.4 μg/ml was used in subsequent experiments.

HELFs were treated with VCR (1.4 μg/ml) for 24 h in the presence or absence of p38 MAPK inhibitor SB203580 or ERK1/2 inhibitor PD98059. It was found that HELF proliferation was markedly inhibited by SB203580 or PD98059. Similar results were obtained by counting the number of cells (F = 62.33, *p* < 0.05 and F = 70.35, *p* < 0.05, respectively) (Figures 1B,D).

### VCR Induces Collagen I Secretion in a Concentration-Dependent Manner

**FIGURE 2:**
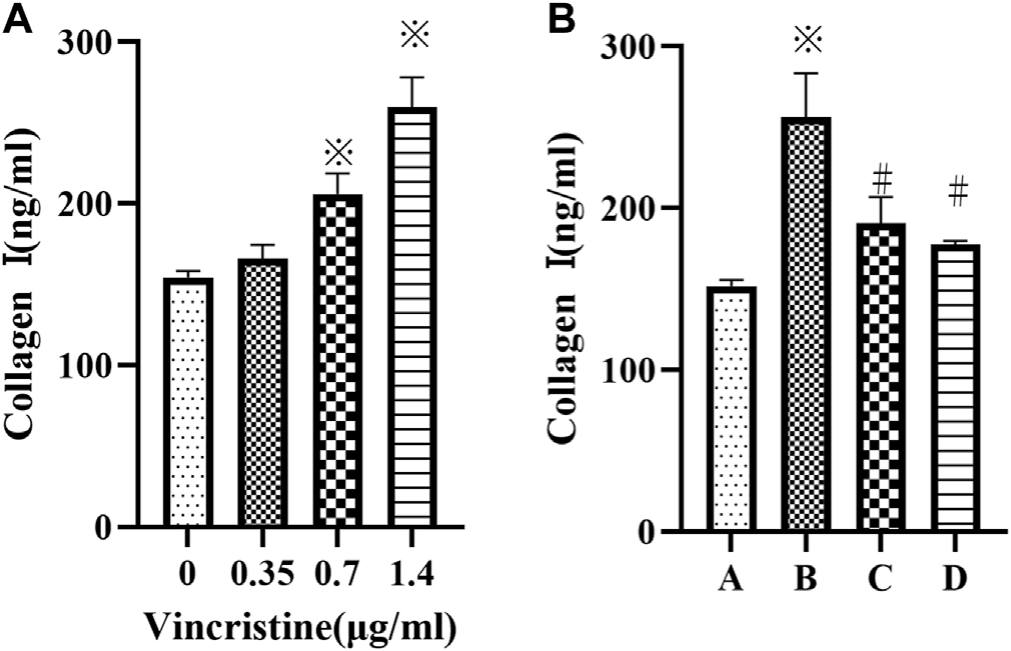
**(A)**: Cells were treated with different concentration of vincristine (0, 0.35, 0.7 and 1.4 μg/ml) for 24 h *p < 0.05 *vs*. the control group. **(B)**: VCR-induced collagen I production was decreased by SB203580 and PD98059 (cells were treated with 1.4 μg/ml VCR for 24 h). We measured the level of collagen I using ELISA assay. *p < 0.05 *vs*. the control group; #*p* < 0.05 *vs*. the VCR group. A: control group, B VCR group, C: SB203580 group, D: PD98059 group.

Compared with the control group, treatment with VCR significantly increased the level of collagen I in a concentration-dependent manner (F = 92.98, *p* < 0.05) (Figure 2A). The most dramatic increase was detected at a concentration of 1.4 μg/ml. Furthermore, the level of collagen I decreased significantly in the presence of SB203580 or PD98059 incubation with 1.4 μg/ml VCR (F = 47.93, *p* < 0.05) (Figure 2B).

### VCR Induces HELF Differentiation *via* p38 and ERK Signal Pathways

**FIGURE 3:**
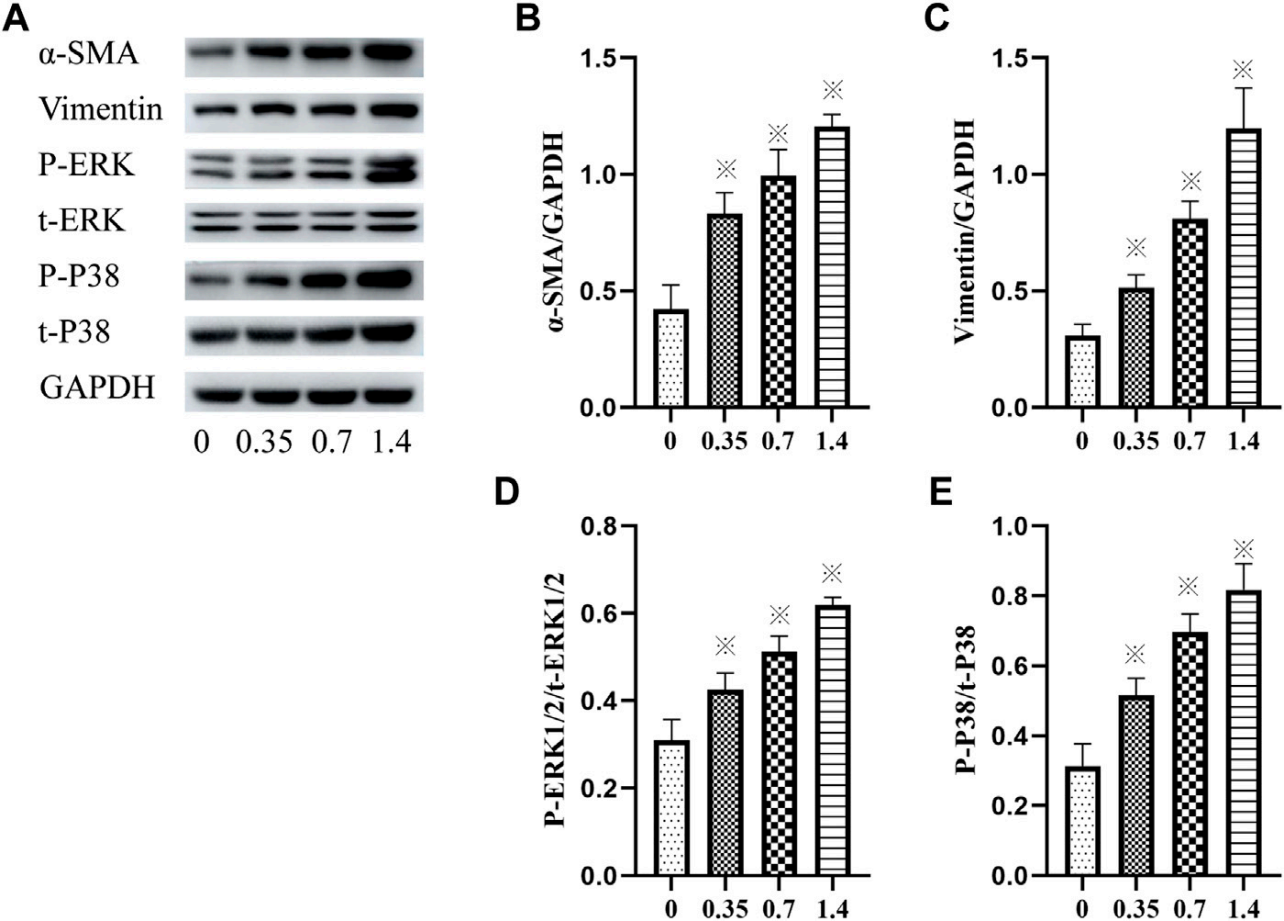
**(A–E)**: *α*-SMA, vimentin, p-ERK1/2, ERK1/2, p-p38 and p38 levels in HELFs were detected using Western blotting. Cells were incubated with different concentration of VCR (0, 0.35, 0.7 and 1.4 μg/ml) for 24 h. Data were presented as mean ± SEM from three independent experiments. *p < 0.05 *vs*. the control group. **(A–D)**: 0,0, 0.35,0.7 and 1.4 μg/ml VCR group.

The expression of vimentin and *α*-SMA was detected using western blotting to confirm lung FB differentiation. The result showed that incubation of HELFs with different concentrations of VCR significantly increased the expression of vimentin and *α*-SMA in a dose-dependent manner. The most significant increase was detected at a concentration of 1.4 μg/ml. (F = 87.93, *p* < 0.05 and F = 78.05, *p* < 0.05, respectively) (Figures 3A–C).

In addition, the phosphorylation and total levels of p38 MAPK and ERK1/2 were detected using western blot analysis. The results revealed that compared with the control group, incubation with VCR significantly increased the amount of p-p38 MAPK, t-p38 MAPK, p-ERK1/2 and t-ERK1/2 in a dose-dependent manner. The most significant increase was detected at a concentration of 1.4 μg/ml. (F = 80.03, *p* < 0.05 and F = 78.20, *p* < 0.05, respectively) (Figures 3A,D,E). Collectively, these findings suggest that VCR induced MB differentiation *via* p38 MAPK and ERK signal pathways.

### Inhibition of p38 MAPK and ERK Signaling Pathways Suppresses VCR-Induced PF

To further determine the mechanism of p38 MAPK and ERK signaling pathways in VCR-induced PF, SB203580 and PD98059 were used to inhibit p38 MAPK and ERK1/2 signaling pathways. Western blot and immunofluorescence assays were used to detect the level of *α*-SMA.

**FIGURE 4:**
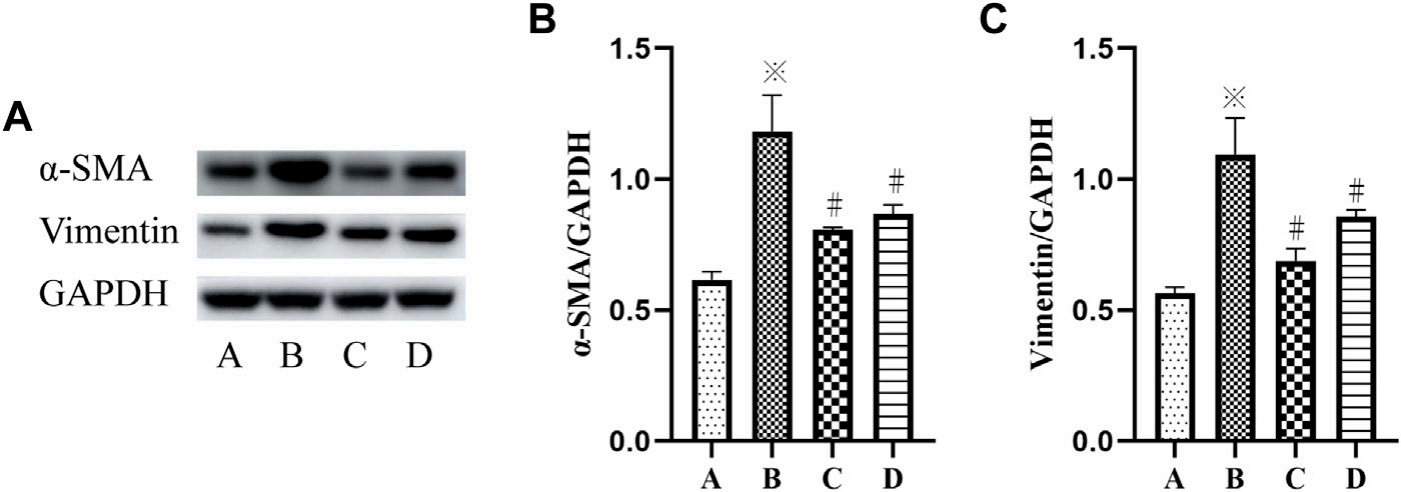
**(A–C)**: *α*-SMA and vimentin levels in HELFs were detected using Western blotting. Cells were which were stimulated with VCR (1.4 μg/ml) for 24 h in presence of SB203580 and PD98059. Data were presented as mean ± SEM from three independent experiments. *p < 0.05 *vs*. the control group; #*p* < 0.05 *vs*. the VCR group. A: control group, B: VCR group, C: SB203580 group, D: PD98059 group.

The results showed a significant increase in the protein expression of vimentin and *α*-SMA in the VCR group (1.4 μg/ml), and the protein expression level of vimentin and *α*-SMA were decreased after SB203580 and PD98059 treatment (F = 55.95, *p* < 0.05 and F = 61.40, *p* <0.05) (Figures 4A–C).

**FIGURE 5:**
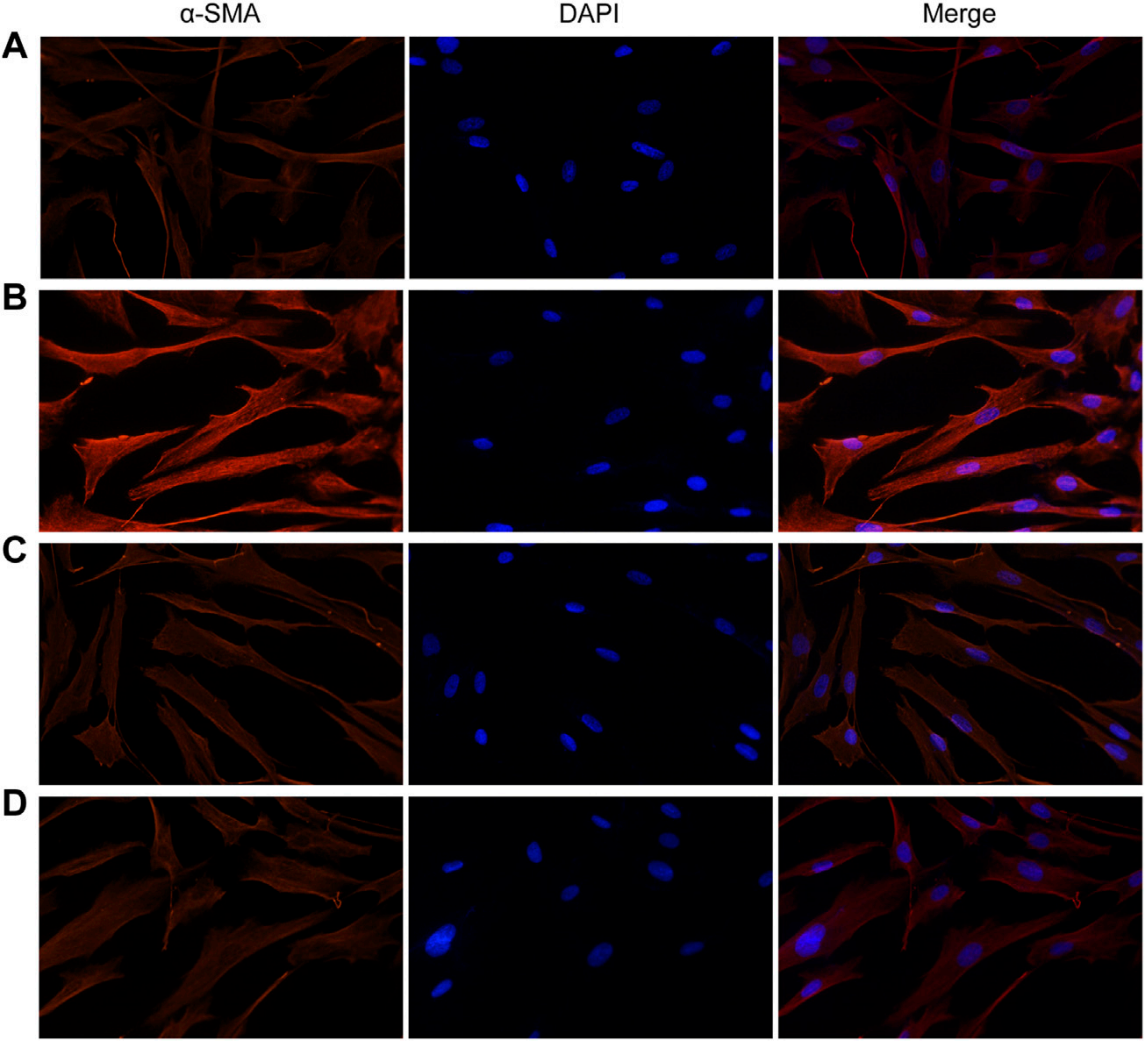
Expression of *α*-SMA induced by VCR in HELFs (original magnification, ×400). HELFs were cultured with VCR (1.4 μg/ml) for 24 h. Immunocytochemical analysis showed that *α*-SMA expression was markedly increased in the VCR group compared with the control group, but was decreased in the two inhibitor groups. Red represents *α*-SMA; blue represents nuclei. **(A)**: control group, **(B)**: VCR group, **(C)**: SB203580 group, **(D)**: PD98059 group.

Similarly, fluorescence analysis results showed that *α*-SMA expression was significantly increased in the VCR group (1.4 μg/ml) compared with the control group (Figure 5). *α*-SMA expression levels were dramatically reduced in groups pretreated with SB203580 and PD98059. Together, these results indicate that p38 MAPK and ERK are involved in the modulatory effects of VCR-induced PF.

## Discussion

While VCR is being widely in the clinic for the treatment of various conditions (such as breast cancer, bronchogenic carcinoma, soft tissue sarcoma and neuroblastoma) (Chao et al., 2015), it may cause several adverse effects in the long term, for instance, lung toxicity, especially PF, which is life-threatening. Moreover, relatively little research has been conducted on molecular mechanisms of VCR-induced PF. Thus, revealing the underlying mechanism of VCR-induced lung injury may help to optimize the treatment strategy and reduce its adverse effect in clinical practice. Studies have shown that the proliferation of MBs is one of the main characteristic features of PF (Huang et al., 2017; J.; Li J et al., 2020). Our study shows that VCR could lead to the transformation of FBs into MBs, revealing the important role of FB proliferation in the pathogenesis of VCR-induced PF.

Pulmonary FBs are important effector cells in the occurrence and development of PF. HELFs have been widely used to study the transdifferentiation of fibroblasts into myofibroblasts and the mechanism of PF *in vitro* (Zhang et al., 2018; Gu et al., 2021). Therefore, we also regard HELFs as fibroblasts in this study. The expression of *α*-SMA is considered to be one of the markers of MBs and a phenotypic marker of FB differentiation (Shinde et al., 2017). In PF, there are a large number of FBs, mainly MBs, accumulating in lung connective tissues, which are speculated to be formed by the transformation of FBs in lung stroma (Pechkovsky et al., 2012; Lin et al., 2020). Dense bundles of *α*-SMA in MB cytoplasm have a contractile function and reduce lung compliance, which is closely related to its fibrogenic function. Vimentin is a member of the intermediate fiber family (Yang et al., 2021). It constitutes the main intermediate fiber in interstitial cells. Its primary function is to form the cell scaffold network with microtubule and microfilaments to maintain their integrity. It plays an important role in cell adhesion, migration, metastasis, proliferation and apoptosis. Vimentin is derived from MBs and endothelial cells. It is also a typical marker of mesenchymal cells. In the present study, we analyzed the specific protein levels of MBs. The expression of *α*-SMA and vimentin was assayed using western blot analysis. It was found that the protein expression of *α*-SMA and vimentin increased significantly after 12-h treatment with VCR. Hence, it was inferred that VCR promotes the development of PF by stimulating the transformation of FBs into MBs.

The mitogen-activated protein kinase (MAPK) is the most important growth regulatory protein. It is the key connection between the cytoplasm and nucleus. It influences the response of cells to changes in the external environment. It can regulate various inflammatory mediators and the expression of cytokines. It plays an important regulatory role in the physiological processes of cell growth, differentiation, development, neural function, immune function, etc. (P. Zhao et al., 2018). It is also reported to play a key role in MB differentiation and ECM deposition.

P38 MAPK, as an important member of the MAPK family, is mainly in the cytoplasm in the resting state. It can be phosphorylated and transferred to the nucleus after stimulation by hypoxia, ultraviolet and other factors. It plays a key role in the regulation of inflammatory response and wound healing by regulating the activity of transcription factors and the synthesis of cytokines (Deng et al., 2021).

The activation of the ERK signaling pathway is associated with many pro-fibrotic cytokines and kinases, such as transforming growth factor-β (TGF-β), platelet-derived growth factor, matrix metalloproteinases, etc. The expression of activated ERK1/2 protein was up-regulated in human or animal models of PF (Liu et al., 2021). The degree of PF was significantly reduced after the administration of inhibitors of ERK signaling pathway PD98059, indicating that this signal pathway is involved in the process of PF. Another study revealed that TGF-β-induced protein expression of human basic fibroblast growth factor (bFGF) could stimulate ERK phosphorylation, which can be inhibited by nonspecific inhibitors of the ERK signaling pathway PD98059. It showed that the ERK signaling pathway plays an important role in MB proliferation, apoptosis and type I collagen proliferation. Limiting the activity of the ERK signaling pathway can inhibit FB proliferation and reduce the production of ECM (Dong et al., 2017).

The current study showed that VCR induced increased protein and gene levels of p-p38 and p-ERK, and P38 and ERK signaling pathways were activated in the cell model of MBs. Our data showed that p38 MAPK and ERK are involved in the modulatory effects of VCR-induced PF.

In summary, for the first time, our study demonstrated that VCR could promote the transformation of FBs into MBs by inducing the phosphorylation of ERK and p38 MAPK. It means that the regulation of ERK and p38 MAPK may provide a target for clinical treatment of VCR-induced PF.

## Data Availability

The original contributions presented in the study are included in the article/Supplementary Materials, further inquiries can be directed to the corresponding authors.

## Abbreviations

α-SMA, A-smooth muscle actin; CCK-8, Cell counting kit -8; DAPI, 4,6-diamidino-2-phenylindole; ECM, Extracellular matrix; ELISA, Enzyme-Linked Immunosorbent Assay; ERK1/2, Extracellular signal-regulated kinase 1/2; FB, Fibroblasts; FCS, Fetal calf serum; helfs, Human Embryonic Lung Fibroblasts; MAPK, Mitogen-activated protein kinase; MB, Myofibroblast; PBS, Phosphate-buffered saline; p-ERK1/2, Phosphorylated ERK1/2; PF, Pulmonary fibrosis; p-p38, Phosphorylated p38 MAPK; RIPA, Radioimmunoprecipitation assay; VCR, Vincristine.
